# Multimodality Monitoring for Delayed Cerebral Ischemia in Subarachnoid Hemorrhage: A Mini Review

**DOI:** 10.3389/fneur.2022.869107

**Published:** 2022-04-13

**Authors:** Collin M. Labak, Berje Haroutuon Shammassian, Xiaofei Zhou, Ayham Alkhachroum

**Affiliations:** ^1^Department of Neurosurgery, Case Western Reserve University School of Medicine, Cleveland, OH, United States; ^2^Department of Neurosurgery, University Hospitals Cleveland Medicine Center, Cleveland, OH, United States; ^3^Department of Neurology, Division of Neurocritical Care, University of Miami Leonard M. Miller School of Medicine, Miami, FL, United States; ^4^Department of Neurology, Jackson Memorial Hospital, Miami, FL, United States

**Keywords:** vasospasm, delayed cerebral ischemia, subarachnoid hemorrhage, monitoring, multimodal monitoring

## Abstract

Aneurysmal subarachnoid hemorrhage is a disease with high mortality and morbidity due in large part to delayed effects of the hemorrhage, including vasospasm, and delayed cerebral ischemia. These two are now recognized as overlapping yet distinct entities, and supportive therapies for delayed cerebral ischemia are predicated on identifying DCI as quickly as possible. The purpose of this overview is to highlight diagnostic tools that are being used in the identification of DCI in the neurocritical care settings.

## Introduction

Aneurysmal subarachnoid hemorrhage (aSAH) is a devastating disease process, with overall mortality approaching 30% (1). Despite significant advances in diagnosis, treatment, and management of SAH, delayed cerebral ischemia (DCI) remains an extremely morbid complication occurring in approximately 30–50% of patients within the first 2 weeks of aSAH (2, 3). The development of DCI significantly impacts prognosis in aSAH patients, leading to persistent focal or cognitive neurological deficit. Treatment for DCI remains challenging; however, a successful strategy begins with early detection and effective monitoring throughout the period of risk.

Although the risk of DCI is associated with the initial volume of cisternal and ventricular hemorrhage, the complex pathophysiology of this disease process is not exclusive to vasospasm (4). This is further evidenced by the presence of infarcts that occur outside of territories demonstrating vessel narrowing on imaging (4). The current definitions of DCI include a clinical component of a decline in GCS of 2 points or greater not attributed to another pathology, or a radiographic component of a cerebral infarction on imaging within 6 weeks of SAH, but not present on imaging within 48 h (5, 6). Neurological examination is the most fundamental modality of identifying patients with DCI, and frequent neurologic assessments are the most accurate modality for detection (7). The examination has a high negative predictive value particularly in patients who are awake, and a good examination will have high accuracy for identifying DCI, with certain studies advocating multiple assessments a day by a neurocritical care provider (7, 8).

However, the most effective monitoring, especially in patients with difficult to ascertain neurological examination, likely includes a combination of modalities that allows for the evaluation of impending temporo-regional ischemia through direct or surrogate measures. The aim of this overview is to highlight both foundational and emerging tools to assist in the diagnosis and monitoring of DCI and vasospasm.

## Methods

A Medline search utilizing PubMed (1966–2021) with a combination of MeSH terms and non-MeSH keywords was conducted. MeSH terms included “cerebral vasospasm,” “imaging, perfusion,” “EEG,” “doppler transcranial ultrasonography,” “microdialysis,” and “computed tomography and single photon emission computerized tomography.” Keywords included “transcranial doppler,” “near-infrared spectroscopy,” “brain-tissue oxygen,” “electroencephalography,” “thermal diffusion flowmetry,” “computed tomography perfusion,” “magnetic resonance perfusion” and “intraparenchymal pressure monitor.” Keywords were combined with the terms “Vasospasm” or “delayed cerebral ischemia.” Abstracts were subsequently reviewed and included for relevance, based on author experience. Pertinent topics identified after full text review were also included when possible. The extent of the topics included in this brief review are mainly limited to those that somehow provide the ability to diagnose DCI and vasospasm (Table 1).

**Table 1:** Multimodal monitoring for delayed cerebral ischemia in subarachnoid hemorrhage.

**Modality**	**Parameter measured**	**Interpretation**	**Benefits**	**Limitations**
EEG	ADR, AP, RAV	↓ADR, AP, RAV with DCI	Available, non-invasive, continuous	Requires qEEG software, experience in interpretation
TCDs	MFV, LR	↑MFV and ↑LR with DCI	Readily available, low cost, non-invasive	Poor inter-operator reliability
Perfusion imaging	CBV, CBF, MTT	Infarct: ↑↑MTT, ↓↓CBF, ↓↓CBV Penumbra: ↑MTT, ↓CBF, normal or ↑CBV	Reliable, differentiates infarcted vs. salvageable tissue, non-invasive	Requires transport to radiology;
Microdialysis	Metabolic markers	↑lactate:pyruvate ratio with DCI	Available with invasive cerebral monitoring kits	Invasive; little data for unique utility; cost of equipment
Jugular venous sampling	SjvO2	↓SjVO2 with DCI	Available, obtained with CVC blood draws	Moderately invasive (Requires central venous catheter)
Cerebral perfusion flowmetry	CBF/perfusion	↓CBF, ↓perfusion with DCI	Available with invasive cerebral monitoring kits	Calibration issues; cost of equipment
Cerebral oxygen monitor	PbtO2	↓PbtO2 with DCI	Available with invasive cerebral monitoring kits	Invasive; cost of equipment

*ADR, alpha/delta ratio; AP, absolute power; CBF, cerebral blood flow; CBV, cerebral blood volume; CVC, central venous catheter; DCI, delayed cerebral ischemia; LR, Lindegaard ratio; MFV, mean flow velocity; MTT, mean transit time; PbtO2, brain tissue oxygenation; qEEG, quantitative electroencephalography; SjVO2, jugular venous oxygen saturation*.

## Modalities

### Transcranial Doppler

Transcranial doppler (TCD) ultrasonography remains the cornerstone of monitoring for vasospasm since its introduction in the early 1980s (9). This modality remains an inexpensive, non-invasive measure of intracranial vessel flow waveforms (10). The flow velocity increases with vasospasm, and thus TCD values can be utilized to assess likelihood and degree of angiographic vasospasm (11). The most definitive utility of the modality to assess vasospasm, with high positive predictive value (PPV), is velocities > 200 cm/s or high negative predictive value (NPV) of velocities < 120 cm/s (12). The 2012 American Heart Association/American Stroke Association guidelines report TCD as a reasonable monitoring modality for development of vasospasm with Level B evidence to support its use, (though they also acknowledge perfusion imaging is likely more valuable in detecting DCI) (13). In addition to these criteria, an increase in flow velocity of 50% also provides evidence of progressive vasospasm (14). Additionally, a sudden drop from a high to low velocity values may be indicate terminally compromised blood flow as opposed to the resolution of vasospasm.

The literature regarding TCD as a predictive tool for DCI is less compelling. A pooled analysis of observational studies established a sensitivity of 90% and NPV of 92%, but a specificity of 71% and PPV of 57% when establishing a middle cerebral artery mean velocity of 120 cm/s as a cut-off (15). However, the severity of TCD indicated vasospasm is associated with DCI within the evaluated vascular territory (16). This evidence confirms the modality as a screening tool but conveys the complexity of DCI as a pathological entity not exclusive to large vessel vasospasm. The addition of the Lindegaard ratio (middle cerebral artery/extracranial internal carotid artery mean velocities) assists in helping to differentiate physiological states of hyperemia and mostly serves to improve overall sensitivity to diagnose of DCI (17).

Other limitations exist in the utilization of TCDs (10). Patient anatomy variances such as hyperostosis or surgical intervention may make it difficult to obtain the necessary sonographic windows. Further, operator and interpreter experience level can create large variability in daily data, thereby making it difficult to obtain a reliable trend. Another limitation is that TCD can only be used on an intermittent basis and is not a continuous monitoring modality. This will likely evolve, however, as evidence by robotic TCD which includes a head strap situated on a patient with automatic adjustments of the ultrasound probe to continuously monitor flow velocities (18). Finally, prediction of vasospasm in the ACA and posterior circulation vessels using MCF is less accurate (11).

TCDs, despite their accessibility, do demonstrate some reliability issues. They should be interpreted as a trend in a patient at risk for vasospasm/DCI and not as isolated values.

### Electroencephalography

Electroencephalography (EEG) provides a non-invasive, continuous, real-time modality useful in monitoring patients especially with high-grade aSAH, and has been increasingly utilized in recent years (19). Loss of fast frequency is one of the earliest signs of ischemia, as seen in aSAH and intraoperative monitoring for carotid revascularization procedures (20, 21). It is especially helpful for monitoring of perfusion as EEG records cortical layers III and V, which are most likely to be affected by perfusion deficits (22). The use of continuous EEG (cEEG) and quantitative EEG (qEEG), or the use of numerical analysis of EEG data to more objectively quantify pattern changes, have been studied to determine both the indicators and predictors of DCI, which is especially useful in contexts where DCI is not due to large-vessel vasospasm, and therefore not adequately assessed *via* angiography or TCDs. Importantly, changes in EEG can also precede vasospasm that can be identified by angiography (23–25). The most common evaluated criteria for qEEG include decreasing alpha/delta ratio (ADR), relative alpha variability, and total power (24, 26). An initial study on qEEG determined a useful marker as an ADR decrease from baseline of either 1) >10% across 6 consecutive readings or 2) >50% on one reading (26). A recent study of 34 patients found those with cerebral infarction demonstrated a greater maximum alpha power decrease and higher number of total hours of alpha power decline, and that maximum TCD frequency was correlated with alpha power reduction (p=0.015) (27). A conducted systematic review indeed found different parameters, including alpha/delta ratio, relative alpha variability, and total power, as having the strongest association depending on the study question (28). In addition to the specific qEEG parameters, patterns on cEEG have also been evaluated in the context of SAH. Although not specific for DCI, changes such as enhanced delta pattern, epileptiform activity, and non-convulsive status epilepticus (NCSE), are all associated with poor outcome across two systematic reviews (28, 29). The benefits of EEG are even greater when combined with TCDs. A recent study identified that utilizing EEG data together with TCD MCA peak systolic velocities was better able to predict DCI than either modality alone (30).

Despite the utility of the advanced capabilities of EEG, the limitations remain in the necessity for the acquisition of both software and hardware, as well as the expertise required in interpreting the acquired data. The availability of this monitoring modality is increasing but still limited, and its cost, though not extreme, is still an added consideration in the use of EEG/qEEG.

### Microdialysis

Microdialysis is a technique used to monitor the extracellular environment of the brain parenchyma as a measure of the metabolic state, and has been increasingly popular within the last two decades as a tool in neurocritical care (31). Clinically, cerebral microdialysis (CMD) probes can be used to measure glucose, lactate, pyruvate, glycerol, and glutamate, with the lactate/pyruvate ratio (LPR) being used as a surrogate for anaerobic metabolism and therefore hypoxic conditions; many groups define a state of metabolic distress as a LPR either >30 or >40, and a glucose <0.7 mmol/L (32, 33). Prior studies have even further correlated cerebral perfusion (CPP) < 70 mmHg to these definitions of metabolic distress, providing evidence of the specific association with blood flow (34).

One of the landmark studies that utilized CMD in aSAH patients was conducted by Veldeman et al. (35). The study was a single-institution analysis of 180 consecutive high-grade aSAH patients divided between the time before and after which the institution implemented invasive neuromonitoring. The authors found an earlier detection of treatable DCI in high-grade aSAH as well as reduction in overall DCI-related infarcts after implementation of invasive neuromonitoring. An additional study that looked specifically at CMD in 28 high-grade aSAH patients in a retrospective fashion found that patients with increased systemic glucose variability were more likely to enter a state of cerebral metabolic distress, which the group defined as a LPR>40 (33). This finding was in turn correlated with in-hospital mortality after adjusting for age, Hunt Hess, daily GCS and symptomatic vasospasm (P = 0.03).

The further benefit of CMD is the ability to demonstrate a predictable change in values prior to the development of radiographic DCI in high grade aSAH patients. Patet et al. showed in comatose patients with DCI the increase in LPR and decrease in glucose over a period of 18 h prior to the development of hypoperfusion on Perfusion Computed Tomography (CPT) (36). Helbok et al. found CMD demonstrated metabolic distress (LPR >40) a median of 13 h prior to the occurrence of corresponding territory infarcts on CT (37).

An important consideration while conducting CMD in the context of pre-existing focal lesions is probe placement given the differential values obtained when evaluating perilesional vs. normal parenchyma. Expectedly, the perilesional microenvironment displays values closer to metabolic impairment more often than normal tissue including lower glucose and higher LPR (38).

Although often associated with invasive neuromonitoring *via* CMD, there has also been work that has assessed jugular bulb microdialysis as a measure that more closely mirrors the cerebral metabolic environment than it does the systemic metabolic environment (39). Although jugular bulb microdialysis in aSAH patients has not been widely assessed, Forsse and colleagues did perform a prospective feasibility study in this patient population, assessing 12 aSAH patients, comparing CMD and jugular bulb microdialysis measurements. They found the method to be generally safe, although various parameters observed between the two measurement devices showed no significant correlations, which suggests that if jugular bulb microdialysis were to be used in aSAH multimodality monitoring, significant legwork would need to be undertaken to determine which metabolic parameters might point to the development of DCI.

### Invasive Brain Tissue Oxygen (PbtO2) and Thermal-Diffusion Flowmetry

Apart from surrogate markers of oxygenation and perfusion, both invasive and non-invasive means are available to evaluate real-time evaluation of changes. As a companion to intraparenchymal pressure monitors, both cerebral oxygen monitors and cerebral thermal-diffusion flowmetry probes have been used in the setting of high-grade subarachnoid hemorrhage (40).

With regard to the evaluation of brain tissue oxygen in subarachnoid patients, there is conflicting evidence as to the benefit on overall clinical outcomes (41, 42). There is some evidence that points to the utility or possible correlation of PbtO2 values and extent of vasospasm, mainly a negative correlation of PbtO2 with degree of angiographic vasospasm (43). A study evaluating the possible association between PbtO2 and TCDs, specifically Lindegaard Ratios, found no correlation (44). However, this study did find a Lindegaard Ratio >/= 3 to have a high specificity for cerebral hypoxia (PbtO2 <20 mmHg) (44). There is also evidence that the combination of these tools may be useful to monitor treatment to refractory vasospasm (45).

A significant utility of PbtO2 monitoring is the ability to combine the modality with CMD. This provides an essential tool with regard to the determination of mitochondrial dysfunction independent of ischemia, demonstrated as an increase in LPR with normal Pyruvate as well as normal PbtO2 values (46). Further, cerebral metabolic distress (LPR > 40) and severe brain tissue hypoxia (PbtO2 < /= 10 mmHg) and more significantly associated together, even in the setting of normal CPP (47).

Cerebral thermal-diffusion flowmetry provides a measure of cerebral blood flow through an intraparenchymal catheter (48). Although much of the literature evaluates the use of this modality in traumatic brain injury, an initial study in patients with anterior circulation aneurysms treated by open surgical clip ligation demonstrated a cutoff value of 15 ml/100 g/min correlated with a sensitivity of 100% and Specificity of 75% of DCI (49). The main limitation of this modality is the highly focal area of perfusion assessment as well as the susceptibility to artifact depending on positioning near vascular structures.

### Near-Infrared Spectroscopy

Although mainly used as an intraoperative monitoring tool in cardiac surgery, near-infrared spectroscopy (NIRS) has gained increasing awareness as a non-invasive modality option within the intensive care unit (50, 51). The modality can display regional cerebral oxygen saturation (rSO2) in the frontal lobes and has previously been validated in stroke patients to correlate with cerebral blood flow through perfusion imaging (52). In high-grade subarachnoid hemorrhage patients, a study by Park et al. found a measurable difference in rSO2 levels in those with DCI compared to those without from days 6–9 as well as an 85.7% sensitivity and specificity for detecting DCI when rSO2 decreased by more than 14.7% (51). Despite this correlation, not all studies have demonstrated an association between NIRS values and symptomatic vasospasm (53).

### Imaging Perfusion Studies

Despite disadvantages with the lack of temporal monitoring, advanced imaging allows for an accurate assessment of territory specific ischemia and infarct, and has been widely implemented in the diagnosis of DCI (54). Any number of the following imaging modalities have been used including Xenon-CT (Xe-CT) (55). Magnetic Resonance Perfusion (MRP) (56). Single-photon emission computed tomography (SPECT) (57, 58) and Perfusion Computed Tomography (PCT) (59).

Among the mentioned techniques, CT perfusion is widely available. The basis to this imaging modality is the formulation of time-density curves at specific regions through dynamic acquisition following a contrast bolus, thus allowing for the evaluation of microcirculation (60). The resultant qualitative maps in conjunction with quantitative values provide a mechanism to evaluate ischemic penumbra and infarct core volumes.

Traditional parameter values that define ischemia or infarct were initially validated in studies of thrombolysis in ischemic stroke. Still, PCT provides a more useful measure than either CTA or CTH alone (61). Prior studies have demonstrated that TTP is the most sensitive parameter for vasospasm, however this is not necessarily in conjunction with DCI as a separate entity (8, 62, 63). Nevertheless, PCT can be utilized to predict clinical outcome in response to endovascular rescue therapy in patients with DCI serving as a potential measure to determine severity (64). Further, PCT can be helpful in diagnosing DCI in patients with poor neurological exams (65).

Perfusion-Weighted MRI evaluates parameters similar to PCT through two available acquisition mechanisms: arterial spin labeling and dynamic susceptibility contrast imaging, the latter which is more commonly utilized (66). Further, Changes in perfusion-weighted MRI parameters correlate well with neurologic deficits in patients with vasospasm (56).

The disadvantages to this technique are those which limit MRI in general, mainly the time required to obtain imaging, which may be pertinent in unstable comatose patients, and the limitations around cardiac devices and metal fragments.

Two additional modalities have been previously used to evaluate perfusion but are not commonly used in the clinical context today: SPECT and Xe-CT. SPECT imaging utilizes the delivery and subsequent uptake of a radioisotope such as technitium-99 m as a corollary to CBF, mainly evaluating relative decreases compared to normally perfused areas. Limitations to this modality include the timely preparation and administration of the radioisotope and the necessity of a normal area of perfusion for analysis (66). Although Xe-CT has been a valid measure to quantify CBF in the past, several limitations prevent this modality from being widely utilized, mainly the need to deliver an inhaled agent, acquisition time, and the susceptibility to artifact from patient motion (66, 67).

### Advanced Hemodynamic Monitoring

Traditional practices of “Triple-H-Therapy” have been supplanted by goal directed approaches to the monitoring and management of both volume status and blood pressure, demonstrating improved outcomes in patients (68). This shift in practice has given rise to literature evaluating novel methods of hemodynamic monitoring in this patient population to more effectively determine cardiac parameters essential for cerebral perfusion (69). Although these hemodynamic parameters do not provide a diagnosis of DCI, the need for continuous and accurate measurements is vital to providing timely care and adjunctive support.

Prior techniques include arterial and central venous pressure monitoring. Two advanced invasive modalities increasingly implemented in the critical care setting include Uncalibrated Pulse Contour Analysis (FloTrac system including the FloTrac sensor and Vigileo Monitor, Edwards, Irvine, CA USA) and calibrated transpulmonary thermodilution (Calibrated TD PiCCO, Pulsion, Munich, Germany and LiDCO Ltd) which allow for the calculation and display of additional hemodynamic parameters specific to the class of device (70).

With regard to the FloTrac system, the obtained values include Stroke Volume (SV), Stroke Volume Variation (SVV), Systemic Vascular Resistance (SVR), and Cardiac Output. In addition to variables obtained from pulse contour analysis, transpulmonary thermodilution provides both Global End Diastolic Volume/Index (GEDV/GEDI) and Extravascular Lung Water Index (ELWI).

The use of transpulmonary thermodilution may elucidate hemodynamic differences in patients with high grade subarachnoid hemorrhage, and more importantly patients with DCI compared to patients without. Yoneda et al. observed a parameter trajectory that generally included a lower GEDI and CI, in conjunction with increased SVRI during the initial half of the vasospasm period in patients with DCI compared to those without (71). Although pooled data do not suggest necessarily a benefit to routine advanced hemodynamic monitoring, the techniques allow for a nuanced and patient specific approach (72).

Finally, there has been some effort placed into assigning risk scores for DCI by analysis of more routinely collected vital sign changes. A recent study by Megjhani and colleagues created a classification/risk stratification model for DCI based on the vital sign data on an hourly basis of 310 aSAH patients (73). Based on the classification they created, when applied to 2 external institutional datasets, they were able to predict 64% and 91% of DCI events as early as 12 h before clinical detection, with 2.7 and 1.6 true alerts for every false alert.

## Conclusion

This brief review provides an overview on the current methods used to assist in the diagnosis and monitoring of DCI and vasospasm. Given the relative advantages and limitations of each modality, the most beneficial approach is a combination of the aforementioned techniques. More importantly, established protocols for the interpretation and subsequent treatment of findings assist in the consistent regimented approach to this complex patient population.

## Author Contributions

CL, BS, XZ, and AA participated in writing the manuscript and editing critical parts of this review. All authors contributed to the article and approved the submitted version.

## Funding

AA is supported by an institutional KL2 Career Development Award from the Miami CTSI NCATS UL1TR002736.

## Conflict of Interest

The authors declare that the research was conducted in the absence of any commercial or financial relationships that could be construed as a potential conflict of interest.

## Publisher's Note

All claims expressed in this article are solely those of the authors and do not necessarily represent those of their affiliated organizations, or those of the publisher, the editors and the reviewers. Any product that may be evaluated in this article, or claim that may be made by its manufacturer, is not guaranteed or endorsed by the publisher.
